# Supplementary material for: Predicting 3D structure and stability of RNA pseudoknots in monovalent and divalent ion solutions
Source: PLoS Comput Biol. 2018 Jun 7;14(6):e1006222. doi: 10.1371/journal.pcbi.1006222 (PMC6007934; doi:10.1371/journal.pcbi.1006222)
Supplement: S1 Text — (PDF) [file pcbi.1006222.s001.pdf]

The force-field of the present model

The total energy U in the present model is composed of following eight components [1,2]

$$U = U_b + U_a + U_d + U_{exc} + U_{bp} + U_{bs} + U_{cs} + U_{el}, \tag{S1}$$

The first three terms are bonded potential for virtual bonds U_b, bond angles U_a and dihedrals U_d, which are used to mimic the connectivity and the local geometry of RNAs.

$$U_b = \sum_{bonds} K_b (r - r_0)^2\,; \tag{S2}$$

$$U_a = \sum_{angles} K_\theta (\theta - \theta_0)^2\,; \tag{S3}$$

$$U_d = \sum_{dihedrals} \{K_\varphi[1 - cos(\varphi - \varphi_0)] + \frac{1}{2} K_\varphi [1 - cos3(\varphi - \varphi_0)]\}, \tag{S4}$$

where K_b, K_θ, and K_φ represent the energy strength; r_0, θ_0, and φ_0 are the distances and angles for virtual bonds, bond angles and dihedrals at energy minimum, respectively. The initial parameters of these three potentials were derived from the Boltzmann inversion of the corresponding atomistic distribution functions obtained by the statistical analysis on the experimental structures in the PDB (http://www.rcsb.org/pdb/home/home.do). To well simulate RNA folding approximated as a free chain, we use the parameters, termed as $\text{Para}_{\text{nonhelical,}}$ which is obtained from the single-strands/loops (nonhelical parts) in experimental structures. In structure refinement, another parameter, termed as $\text{Para}_{\text{helical}}$, which is obtained from the stems (helical parts) in experimental structures, is also used for base paired nucleotides of the native-like structure predicted from the folding process.

U_{exc} in Eq. S1 represents the excluded volume interaction between the CG beads and it is modeled by a purely repulsive Lennard-Jones potential:

$$U_{exc} = \sum_{i<j}^{N} \begin{cases} 4\varepsilon \left[\left(\frac{\sigma_0}{r_{ij}} \right)^{12} - \left(\frac{\sigma_0}{r_{ij}} \right)^{6} \right] & if\ r_{ij} \le \sigma_0 \\ 0 & if\ r_{ij} > \sigma_0 \end{cases}, \tag{S5}$$

where ε = 0.26 kcal/mol is the interaction strength, σ_0 is the sum of the radii of bead i and j, and r_{ij} is the distance between bead i and j.

U_{bp} in Eq. S1 is the orientation-dependent base-pairing interaction between bases in the canonical Watson-Crick base pairs (G-C and A-U) and the wobble base pairs (G-U). The potential is

given by

$$U_{bp} = \sum_{i<j-3}^{N_{bp}} \frac{\varepsilon_{bp}}{1 + k_{NN}\left(r_{N_iN_j} - r_{NN}\right)^2 + k_{CN}\sum_{i(j)}\left(r_{C_iN_j} - r_{CN}\right)^2 + k_{PN}\sum_{i(j)}\left(r_{P_iN_j} - r_{PN}\right)^2}, \quad \text{(S6)}$$

where ε_{bp} is the interaction strength $\varepsilon_{AU} = \varepsilon_{GU} = 0.5\varepsilon_{GC}$ and $\varepsilon_{GC} = -3.5$ kcal/mol in the present model. r_{NN}, r_{CN}, and r_{PN} are three distances between the corresponding atoms of P, C and N in two paired nucleotides to determine whether the base-pair is well formed, and k_{NN}, k_{CN} and k_{PN} are the corresponding energy strength.

U_{bs} in Eq. S1 is the base-stacking interaction between two nearest neighbour base pairs, and the energy is given by

$$U_{bs} = \frac{1}{2}\sum_{i,j}^{N_{st}} |G_{i,i+1,j-1,j}| \left\{\left[5\left(\frac{\sigma_{st}}{r_{i,i+1}}\right)^{12} - 6\left(\frac{\sigma_{st}}{r_{i,i+1}}\right)^{10}\right] + \left[5\left(\frac{\sigma_{st}}{r_{j,j-1}}\right)^{12} - 6\left(\frac{\sigma_{st}}{r_{j,j-1}}\right)^{10}\right]\right\}, \quad \text{(S7)}$$

where σ_{st} is the optimum distance of two neighbour bases in the known helix structures. $G_{i,i+1,j-1,j}$ in Eq. S7 is the strength of base-stacking energy and can be estimated from the combination of the experimental thermodynamics parameters [3] and Monte Carlo simulations:

$$G_{i,i+1,j-1,j} = \Delta H - T(\Delta S - \Delta S_c); \quad \text{(S8)}$$

more details can be found in our related works [1,2].

U_{cs} in Eq. S1 is the coaxial-stacking between the base-pairs which are the interfaces of two discontinuous neighbour helices:

$$U_{cs} = \frac{1}{2}\sum_{i-j,k-l}^{N_{cst}} |G_{i,k,l,j}| \{[1 - e^{-a(r_{ik}-r_{cs})}]^2 + [1 - e^{-a(r_{jl}-r_{cs})}]^2 - 2\}. \quad \text{(S9)}$$

$G_{i,k,l,j}$ is the sequence-dependent base-stacking strength, which is approximately taken as the stacking strength between the corresponding nearest-neighbour base-pairs in an uninterrupted helix [2,3]. r_{ik} (or r_{jl}) is the distance between two interfaced bases $i(j)$ and $k(l)$ of two stems and a represents the extent of distance constraint. r_{cs} is the optimum distance between two coaxially stacked stems, which is directly obtained from the statistical analysis on the known structures in PDB.

U_{el} in Eq. S1 is the electrostatic interaction between phosphates with reduced charges given by the Debye-Hückel approximation [1,4]:

$$U_{el} = \sum_{i<j}^{N_P} \frac{(Qe)^2}{4\pi\varepsilon_0\varepsilon(T)r_{ij}} \mathrm{e}^{-\frac{r_{ij}}{l_D}}. \quad \text{(S10)}$$

where e is the elementary charge and N_P is the number of phosphate beads in an RNA. l_D is Debye

length, which defines the ionic screening. Based on the counterion condensation theory [4] and the tightly bound ion model [5-7], the reduced charge fraction Q could be written as $Q = \frac{b}{vl_B}$ for pure ion monovalent (v=1) or divalent (v=2) ion solutions and $Q = f_{\mathrm{Na}^+}\left(\frac{b}{l_B}\right) + (1 - f_{\mathrm{Na}^+})\left(\frac{b}{2l_B}\right)$ for mixed Na^+/Mg^{2+} solutions, where b is the charge spacing on RNA backbone, l_B is the Bjerrum length, f_{Na^+} and 1-f_{Na^+} are the contribution fraction from Na^+ and Mg^{2+}, respectively. The empirical formula

$$f_{\mathrm{Na}^+} = \frac{[\mathrm{Na}^+]}{[\mathrm{Na}^+] + x[\mathrm{Mg}^{2+}]} \qquad \text{(S11)}$$

derived by the tightly bound ion model is used for mixed divalent/monovalent ion solutions, and $x = (8.1 - 64.8/N)(5.2 - \ln[\mathrm{Na}^+])$, where $[Na^+]$ and $[Mg^{2+}]$ are the corresponding concentrations in molar (M) and N is the chain length [5,7].

The parameters of the above described potentials listed in Table A are derived through the statistical analysis on the known structures and the comparisons between the predictions by the model and the experimental data (see more details in Ref. 1). All the parameters are the same as the previous version of our model [1,2].

Table A. The parameters of potentials in Eqs. S2-S10.

	Bond U_b			
	K_b (kcal/mol/Å^2)		r_0 (Å)	
	$\text{Para}_{\text{helical}}$ [a]	$\text{Para}_{\text{nonhelical}}$ [b]	$\text{Para}_{\text{helical}}$ [a]	$\text{Para}_{\text{nonhelical}}$ [b]
P_iC_i	133.4	98.2	3.95	3.95
C_iP_{i+1}	75.0	42.5	3.93	3.93
C_iN_i	85.6	24.8	3.35	3.45
	Angle U_a			
	K_θ (kcal/mol/rad^2)		θ_0 (rad)	
	$\text{Para}_{\text{helical}}$ [a]	$\text{Para}_{\text{nonhelical}}$ [b]	$\text{Para}_{\text{helical}}$ [a]	$\text{Para}_{\text{nonhelical}}$ [b]
$P_iC_iP_{i+1}$	18.3	9.3	1.74	1.75
$C_{i-1}P_iC_i$	43.9	21.3	1.76	1.78
$P_iC_iN_i$	35.5	9.7	1.63	1.64
$N_iC_iP_{i+1}$	99.8	15.2	1.66	1.66
	Dihedral U_d			
	K_φ (kcal/mol/rad^2)		φ_0 (rad)	
	$\text{Para}_{\text{helical}}$ [a]	$\text{Para}_{\text{nonhelical}}$ [b]	$\text{Para}_{\text{helical}}$ [a]	$\text{Para}_{\text{nonhelical}}$ [b]
$P_iC_iP_{i+1}C_{i+1}$	2.8	1.1	2.56	2.51
$C_{i-1}P_iC_iP_{i+1}$	10.5	4.3	-2.94	-2.92
$C_{i-1}P_iC_iN_i$	3.8	0.8	-1.16	-1.18
$N_{i-1}C_{i-1}P_iC_i$	4.2	0.7	0.88	0.78
	Nonbonded			
r_{NN} (Å)	8.9	k_{NN} (Å^{-2})		3.6
r_{CN} (Å)	12.2	k_{CN} (Å^{-2})		1.9
r_{PN} (Å)	13.9	k_{PN} (Å^{-2})		0.7
σ_{st} (Å)	4.8	ε_{bp} (kcal/mol)		-3.5
r_{cs} (Å)	5.0	a (kcal/mol//Å)		0.4

[a] The $\text{Para}_{\text{helical}}$ are only used in the processes of folded structure refinement for the base-pairing regions (stems) in the initially folded structure. [b] The $\text{Para}_{\text{nonhelical}}$ are used in RNA folding processes to possibly describe RNAs as free chains.

Table B. The 17 RNA pseudoknots for 3D structure prediction used in this work.

RNAs[a]	PDB ID	Length (nt)	L_Stem 1[b] (bp)	L_Stem 2[c] (bp)	L_Loop 1[d] (nt)	L_Loop 2[e] (nt)	L_Loop 3[f] (nt)
1	2g1w	22	4	3	1	5	1
2	1a60	23	3	5	4	3	0
3	2a43	26	4	3	2	9	1
4	2rp1	27	5	3	2	8	1
5	1l2x	28	5	3	2	7	1
6	1kpy	28	5	3	2	8	1
7	1yg4	28	5	3	2	9	1
8	437d	28	5	3	2	7	1
9	2ap5	28	5	3	2	9	1
10	2n6q	31	5	7	2	5	0
11	1kpd	32	5	6	2	7	1
12	1rnk	34	5	7	1	8	1
13	1e95	36	6	6	1	9	0
14	2tpk	36	5	7	1	7	0
15	1ymo	47	6	9	8	8	0
16	2m8k	48	5	10	6	9	2
17	2lc8	56	11[g]	7	1	13	0

[a] For each RNA pseudoknot, PDB ID, chain length and the length of two stems (Stem 1 and Stem 2) and three loops (Loop 1, Loop 2 and Loop 3) are listed. [b,c,d,e,f] The length of Stem 1, Stem 2, Loop 1, Loop 2, and Loop 3 (see Fig. 1 in the maintext), respectively. [g] Stem 1 of the pseudoknot (PDB ID: 2lc8) is interrupted by a bulge and an internal loop.

Table C. The RMSDs of 3D structures of RNA pseudoknots predicted by the present model with and without the coaxial stacking potential.

RNAs	PDB ID	Length (nt)	Coaxial stacking in experimental structures [a]	Mean RMSD [b] (Å)	
				The present model with U_{cs}	The present model without U_{cs}
1	2g1w	22	Yes	5.3	6.2
2	1a60	23	Yes	3.6	4.3
3	2a43	26	No	4.2	**3.9**
4	2rp1	27	No	4.2	4.2
5	1l2x	28	No	5.1	**4.5**
6	1kpy	28	No	4.3	4.4
7	1yg4	28	No	5.4	**5.1**
8	437d	28	No	5.2	**4.5**
9	2ap5	28	No	5.6	**5.1**
10	2n6q	31	Yes	6.4	6.9
11	1kpd	32	Yes	5.9	6.0
12	1rnk	34	Yes	5.9	6.2
13	1e95	36	Yes	5.4	6.5
14	2tpk	36	Yes	4.4	5.7
15	1ymo	47	Yes	9.3	9.9
16	2m8k	48	Yes	8.9	9.7
17	2lc8	56	Yes	7.4	8.2
Mean value				5.6	6.0

[a] For each pseudoknot, is the coaxial stacking between two stems observed in experimental structure? [b] The mean RMSDs for 17 RNA pseudoknots between structures predicted by the present model with and without the coaxial stacking potential and the native structures.

Table D. The melting temperatures (T_{m1} and T_{m2})[a] of six RNA pseudoknots at high salt predicted by the present model with/without the coaxial stacking potential (U_{cs}).

RNA pseudoknots[b]	Coaxial stacking in experimental structures [c]	Expt. (°C) T_{m1}/T_{m2}	The model with U_{cs} T_{m1}/T_{m2} (°C)	The model without U_{cs} T_{m1}/T_{m2} (°C)
MMTV	Yes	73.5/95.0	71.7/96.3	69.1/95.4
T2	Yes	68.9/77.6	67.2/78.8	63.0/78.0
PEMV-1	No	60.1/79.1	54.5/80.8	46.2/79.8
BWYV	No	69.4/91.2	65.8/93.1	54.9/92.4
PLRV	No	67.4/87.5	66.3/85.3	59.3/85.6
ScYLV	No	67.5/77.9	66.2/80.2	61.9/79.8

[a] T_{m1} and T_{m2} are the melting temperatures for the transitions from folded (pseudoknot) state to intermediate (hairpin) state and from intermediate state to unfolded (coil) state, respectively. [b] MMTV and T2 pseudoknots are at 1000 mM [K^+]; PEMV-1, BWYV, PLRV and ScYLV pseudoknots are at 500 mM [K^+]. [c] For each pseudoknot, is the coaxial stacking between two stems observed in experimental structure?

Supporting References

1. Shi YZ, Wang FH, Wu YY, Tan ZJ. A coarse-grained model with implicit salt for RNAs: predicting 3D structure, stability and salt effect. J. Chem. Phys. 2014; 141: 105102.
2. Shi YZ, Jin L, Wang FH, Zhu XL, Tan ZJ. Predicting 3D structure, flexibility, and stability of RNA hairpins in monovalent and divalent ion solutions. Biophys. J. 2015; 109: 2654-2665.
3. Xia T, SantaLucia J, Burkand ME, Kierzek R, Schroeder SJ, Jiao X, Cox C, Turner DH. Thermodynamic parameters for an expanded nearest-neighbor model for formation of RNA duplexes with Watson-Crick base pairs. Biochemistry 1998; 37: 14719-14735.
4. Manning GS. The molecular theory of polyelectrolyte solutions with applications to the electrostatic properties of polynucleotides. Q. Rev. Biophys. 1978; 11: 179-246.
5. Tan ZJ, Zhang W, Shi YZ, Wang FH. RNA folding: structure prediction, folding kinetics and ion electrostatics. Adv. Exp. Med. Biol. 2015; 827: 143-183.
6. Tan ZJ, Chen SJ. Electrostatic correlations and fluctuations for ion binding to a finite length polyelectrolyte. J. Chem. Phys. 2005; 122: 044903.
7. Tan ZJ, Chen SJ. RNA helix stability in mixed Na^+/Mg^{2+} solution. Biophys. J. 2007; 92: 3615–3632.
