# Supplementary figures and images for: Predicting 3D structure and stability of RNA pseudoknots in monovalent and divalent ion solutions
Source: PLoS Comput Biol. 2018 Jun 7;14(6):e1006222. doi: 10.1371/journal.pcbi.1006222 (PMC6007934; doi:10.1371/journal.pcbi.1006222)

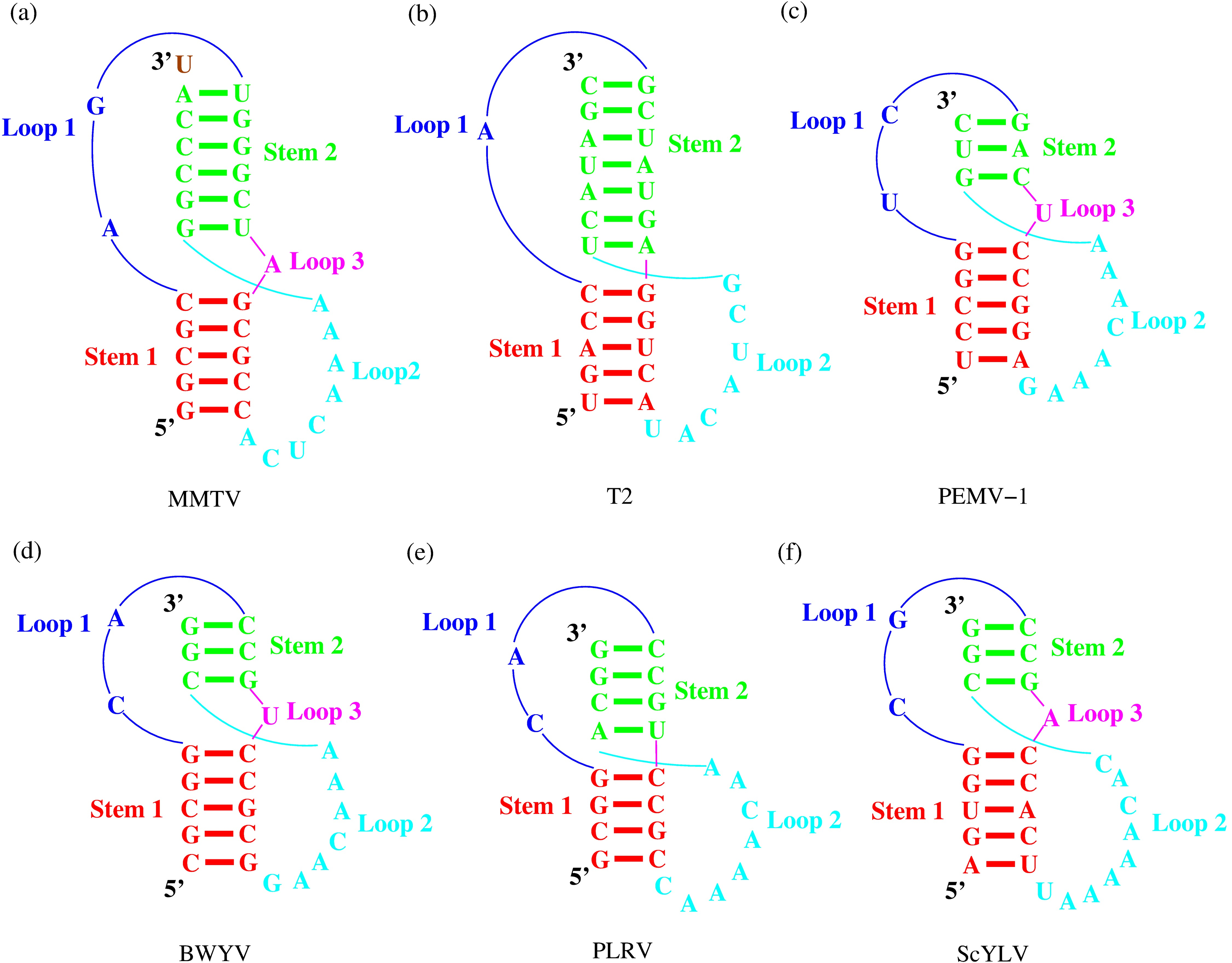

Supplement: S1 Fig — (a) MMTV pseudoknot; (b) T2 pseudoknot; (c) PEMV-1 pseudoknot; (d) BWYV pseudoknot; (e) PLRV pseudoknot; (f) ScYLV pseudoknot. (TIF) [file pcbi.1006222.s002.tif]

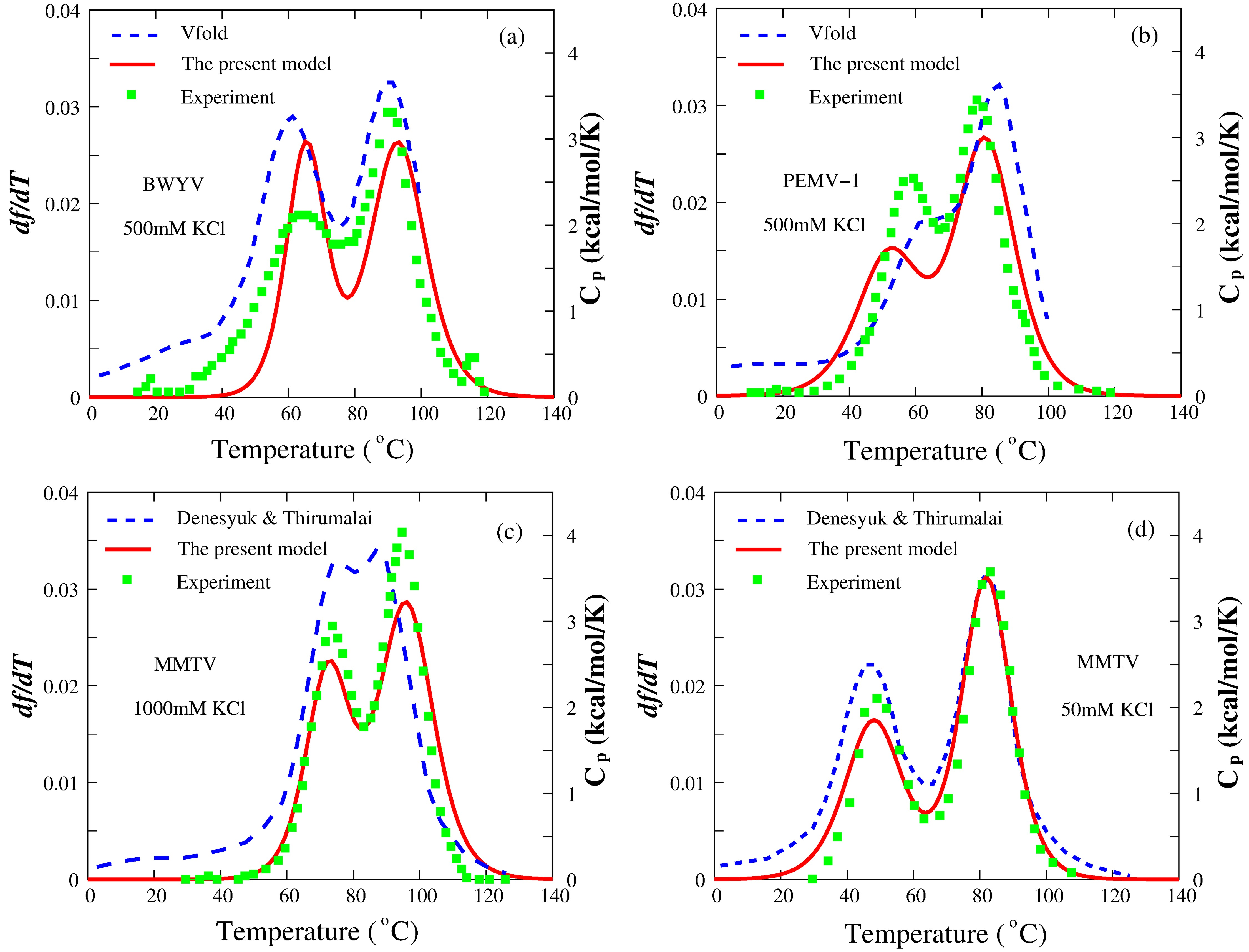

Supplement: S2 Fig — (a,b) BWYV (a) and PEMV-1 (b) pseudoknots at 500mM [K+], respectively. Solid lines: df/dT, the first derivative of f with respect to temperature from the present model. Dotted lines: the heat capacity Cp from Ref. 78. Symbols: the heat capacity Cp from experiments [72,73]. (c,d) MMTV pseudoknot at 1000mM [K+] (c) and 50mM [K+] (d), respectively. Solid lines: df/dT, the first derivative of f with respect to temperature from the present model. Dotted lines: the heat capacity Cp from Ref. 46. Symbols: the heat capacity Cp from experiments [75]. (TIF) [file pcbi.1006222.s003.tif]

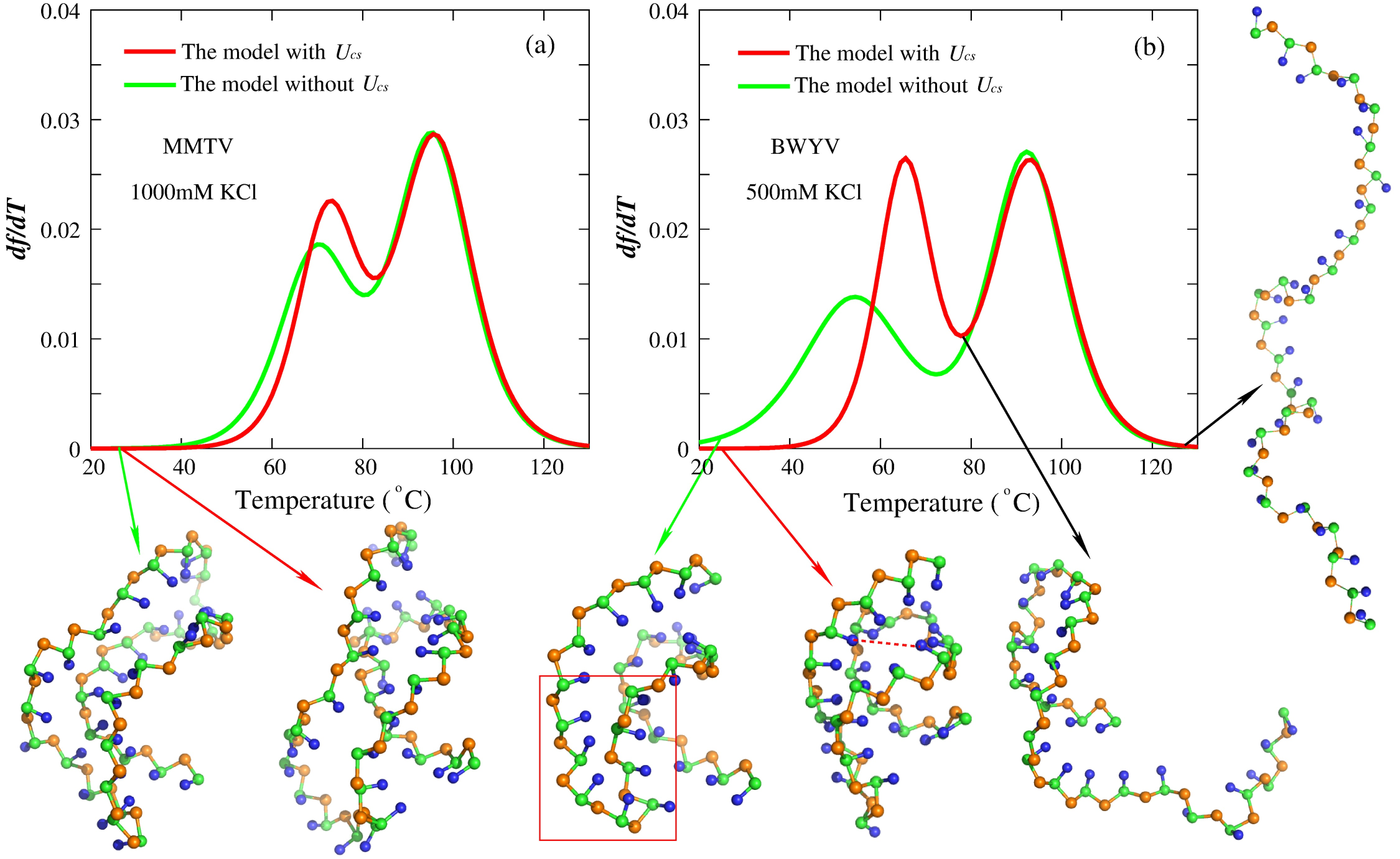

Supplement: S3 Fig — (a) MMTV pseudoknot at 1000mM [K+]; (b) BWYV pseudoknot at 500mM [K+]. Lines: df/dT, the first derivative of f with the temperature; red: predictions from the model with the coaxial stacking potential; green: predictions from the model without the coaxial stacking potential. Cartoon: the predicted 3D structures of the two pseudoknots at different temperatures. Red/black arrow: predictions from the model with coaxial stacking potential; green arrow: predictions from the model without the coaxial stacking potential. (TIF) [file pcbi.1006222.s004.tif]

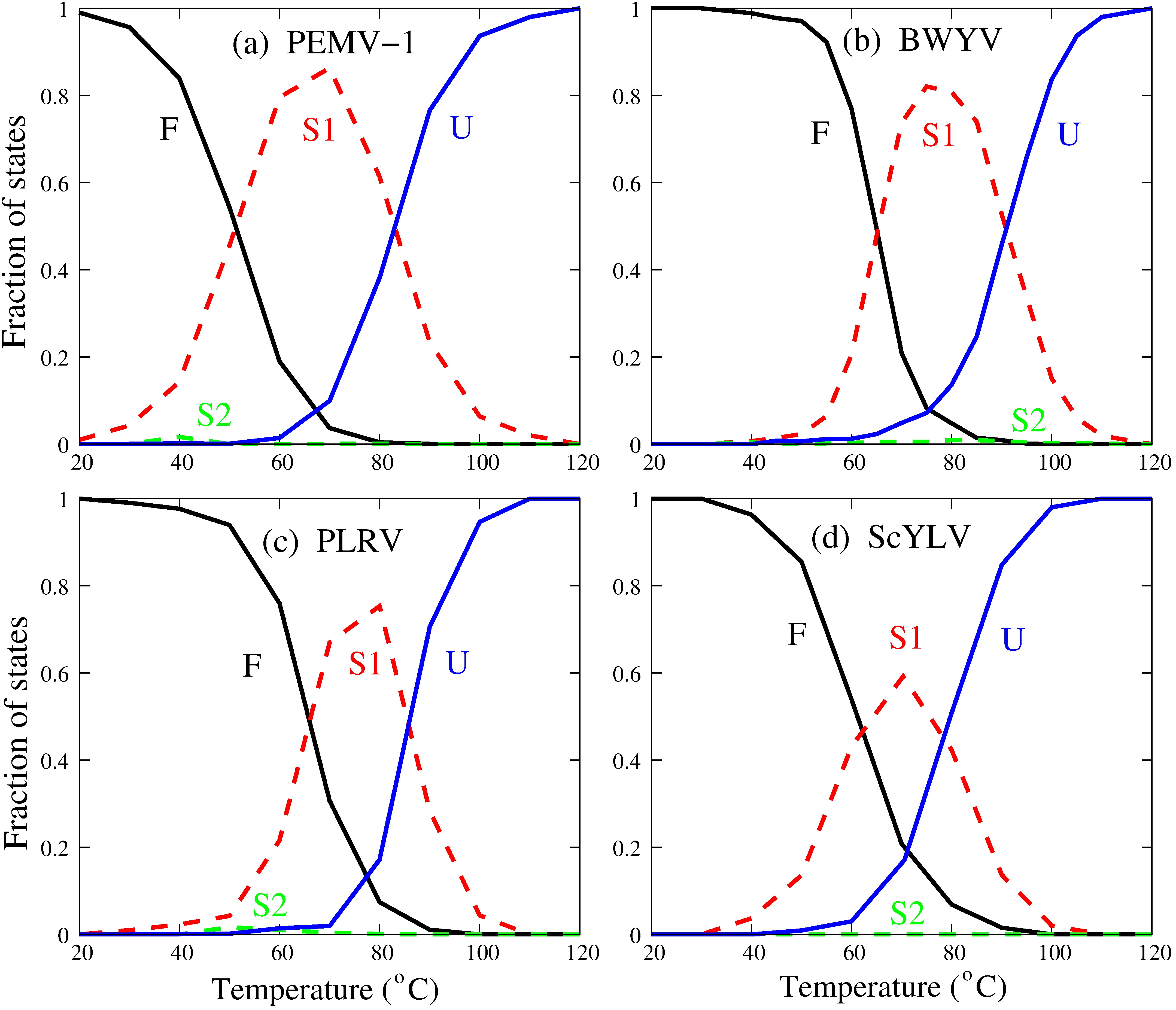

Supplement: S4 Fig — (a) PEMV-1, (b) BWYV, (c) PLRV, and (d) ScYLV pseudoknots. F stands for fully folded RNA; S1, hairpin intermediate with Stem1; S2, hairpin intermediate with Stem2; U, fully unfolded RNA. (TIF) [file pcbi.1006222.s005.tif]

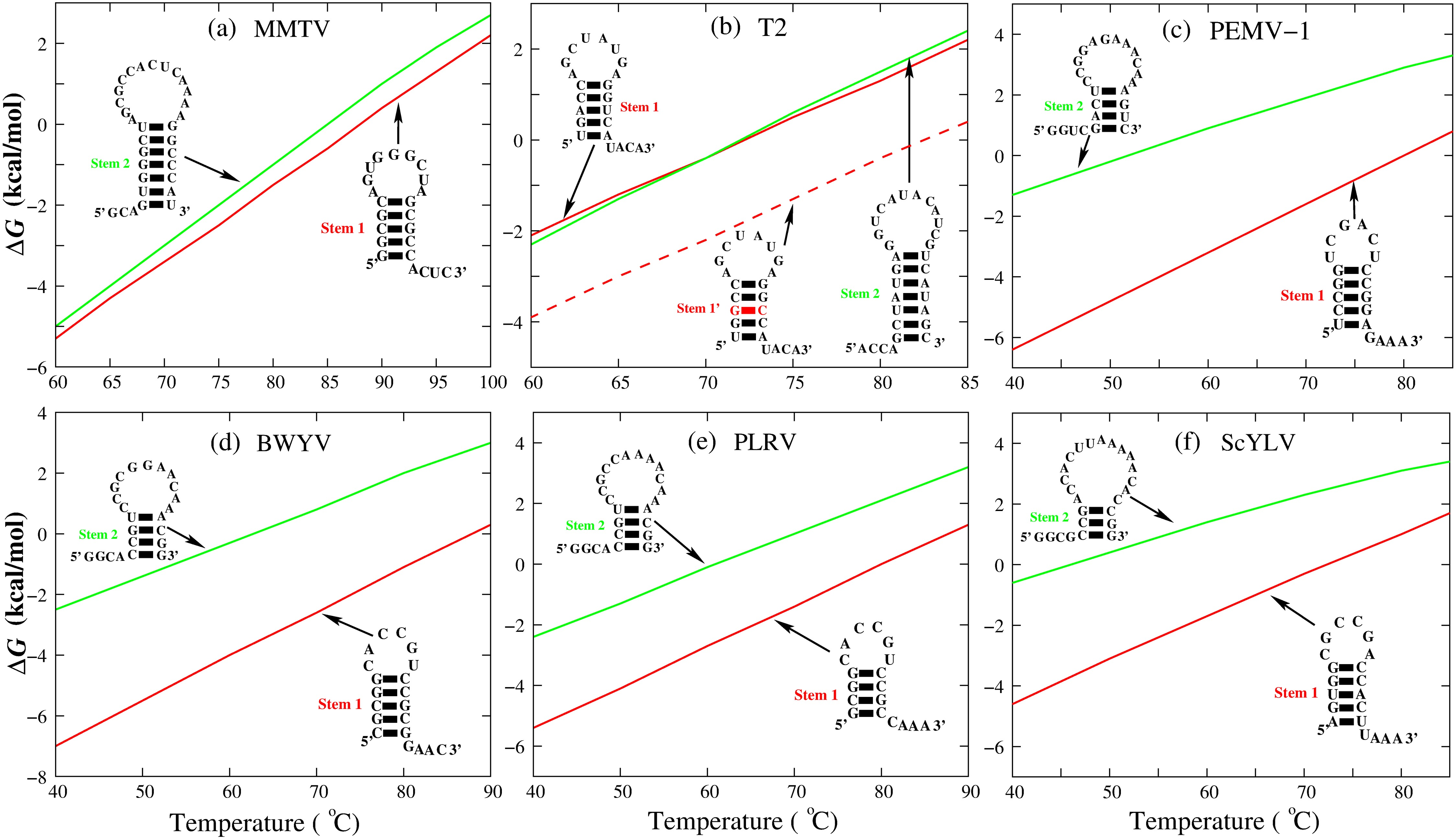

Supplement: S5 Fig — (a) MMTV pseudoknot; (b) T2 and T2 variant pseudoknots; (c) PEMV-1 pseudoknot; (d) BWYV pseudoknot; (e) PLRV pseudoknot; and (f) ScYLV pseudoknot. Here, the free energies are computed using Mfold (http://unafold.rna.albany.edu/) [89]. (TIF) [file pcbi.1006222.s006.tif]

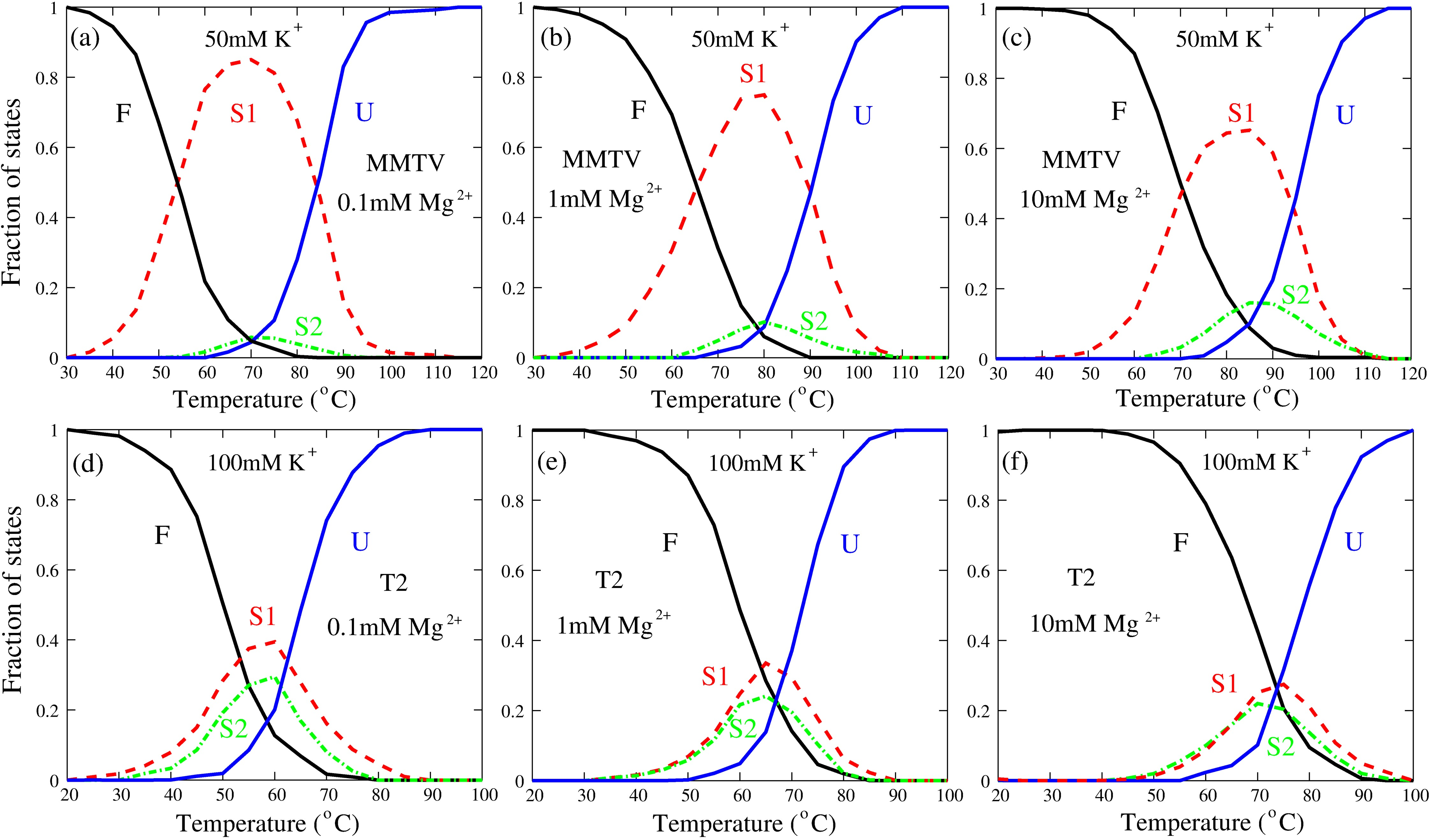

Supplement: S6 Fig — (a-c) MMTV pseudoknot at 50mM [K+] and different [Mg2+]’s: (a) 0.1mM [Mg2+], (b) 1mM [Mg2+], and (c) 10mM [Mg2+]. (d-f) T2 pseudoknot at 100mM [K+] and different [Mg2+]’s: (d) 0.1mM [Mg2+], (e) 1mM [Mg2+], and (f) 10mM [Mg2+]. (TIF) [file pcbi.1006222.s007.tif]
